# Methods of determing biokinetics of arsenate accumulation and release in *Microcystis aeruginosa* regulated by common environmental factors: Practical implications for enhanced bioremediation

**DOI:** 10.1016/j.mex.2018.08.010

**Published:** 2018-08-28

**Authors:** Zhenhong Wang, Zhuanxi Luo, Changzhou Yan, Ricki R. Rosenfeldt, Frank Seitz, Herong Gui

**Affiliations:** a Key Laboratory of Urban Environment and Health, Institute of Urban Environment, Chinese Academy of Sciences, Xiamen, 361021, China; b College of Chemistry and Environment and Fujian Province Key Laboratory of Modern Analytical Science and Separation Technology, Minnan Normal University, Zhangzhou, 363000, China; c Institute for Environmental Sciences, University of Koblenz-Landau, Fortstrasse 7, 76829, Landau, Germany; d nEcoTox, An der Neumuehle 2, 76855, Annweiler, Germany; e National Engineering Research Center of Coal Mine Water Hazard Controlling (Suzhou University), Suzhou, Anhui, 234000, China

**Keywords:** Taguchi experimental design for arsenic metabolic biokenetics, Algal bioremediation, Accumulation, Release, Dead algae, Taguchi

## Abstract

Only little information is available on combined effects of abiotic environmental factors on algal arsenate (*As^V^*) metabolic biokinetics. Herein, we demonstrated the methods of using the Taguchi statistical method to investigate four environmental factors including *As^V^*, nitrate (N), orthophosphate (P) and pH for their combined effects on algal growth and arsenic (*As*) uptake but also extracellular adsorption of *Microcystis aeruginosa,* as well as *As* release from dead algal cells. Results showed that an increase of N facilitated *M. aeruginosa* growth and thus was the principal factor for the algal maximum specific growth rate ($\;\mu_{\text{max}}$). P was vital to *As^V^* bioconcentration factor (BCF) and *As* partition coefficients (Log*K_d_*) released from deal algal cells. *As^V^* impacted the extracellular *As* adsorption onto the algal cells, which thereby increased with increasing initial *As^V^* level. The initial pH had an imperative effect on the *As^V^* uptake (*k_u_*) and release rate (*K_e_*) from the dead cells. Collectively, the condition of low P, high N and alkaline pH level was favorable to *As* accumulation rate of living cells and restrictive to *As* release rate from dead cells of *M. aeruginosa*. The obtained information can pave a road for extensive understanding on efficient utilization of *As* bioremediation of algae in practical environment.

• Principal factors were identified on *As^V^* metabolic biokinetics by Taguchi method.
• High N and pH but low P fasten *As^V^* uptake and reduce *As* efflux from dead cells.
• *As^V^* only as the main factor impacted *As* extracellular adsorption on algal cells.

## Graphical abstract

**Figure:**
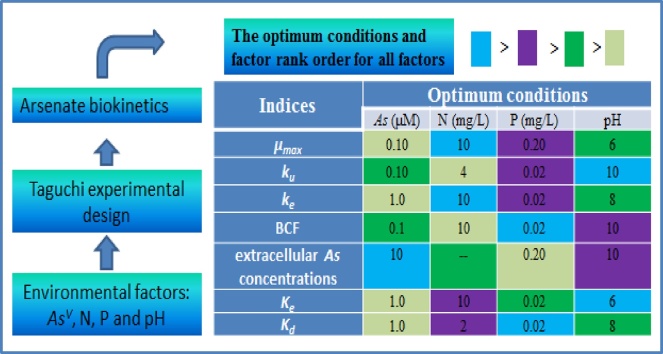

**Specifications Table**

**Table:** 

Subject area	*Select one of the following subject areas:* • *Agricultural and Biological Sciences* • *Earth and Planetary Sciences* • *Engineering* • *Environmental Science*
More specific subject area	*Arsenic bioremediation by algae in water*
Method name	Taguchi Experimental design for arsenic metabolic biokenetics.
Name and reference of original method	–
Resource availability	–

## Method details

### Experimental design

The Taguchi method, used to optimize the experimental design, is the same being applied during our previous study [1]. Briefly, four common environmental factors including *As^V^*, N, P and pH were considered each at three levels (Table 1) [2]. The detailed experimental conditions were obtained using a L9 (3^4^) orthogonal array (Table 2). Furthermore, we used bigger analysis values of signal-to-noise (S/N) ratio to assess optimal conditions for *As* metabolic biokinetics in *M. aeruginosa*. Additionally, the principal contribution factors for various *As* metabolic biokinetic parameters were evaluated using ANOVA in *M. aeruginosa*. The software of Minitab 17 was used to perform the statistical analysis of the following data obtained.

**Table 1:** Environmental factors of orthogonal test.

	factor			
	A NO_3_^−^-N/(mg/L)	B PO_4_^3−^-P/(mg/L)	C pH	D *As^V^*/(μM)
level 1	2	0.02	6	0.1
level 2	4	0.20	8	1.0
level 3	10	1.00	10	10.0

**Table 2:** Experimental L_9_ (3^4^) orthogonal array.

Treatment	Parameters			
	NO_3_^−^-N/(mg/L)	PO_4_^3−^-P/(mg/L)	pH	*As^V^*/μM
E1	2	0.02	6	0.1
E2	2	0.2	8	1.0
E3	2	1.0	10	10
E4	4	0.02	8	10
E5	4	0.2	10	0.1
E6	4	1.0	6	1.0
E7	10	0.02	10	1.0
E8	10	0.2	6	10
E9	10	1.0	8	0.1

### *M. aeruginosa* culture growth

Stock cultures *of M. aeruginosa* (FACHB-905) were maintained in sterilized BG-11 media on shakers at 90 rpm (25 °C) under a 8:16 h dark-light cycle with a light intensity of 40 μmol photons m^−2^ s^−1^. We prepared the nine BG-11 media under the Taguchi designed experimental conditions (Table 2). As N, *As^V^* and P source, stock solutions of 1000 mg L^−1^ NO_3_^−^-N from NaNO_3_ as well as 1000 mg L^−1^ *As^V^* from Na_3_AsO_4_·12H_2_O (Fluka, p.a.), and 100 mg L^−1^ PO_4_^3−^-P from KH_2_PO_4_ were prepared. Additionally, respective pH values were adjusted in the media using 1 M NaOH or 1 M H_2_SO_4_ at the start of each experiment.

### Arsenic metabolic biokinetics

#### Uptake experiment and kinetics model

Firstly, *M. aeruginosa* cells were separated into nine equal parts after starving cultures. Then they were aseptically transferred to nine different sterilized BG-11 media with an initial cell density of 10^6^ cells mL^−1^ applying the Taguchi designed experimental conditions (Table 2). The batch treatments were cultured in an illuminated incubator which was permanently shaking for 96 h. After 3, 24, 48, 72 and 96 h, approximately 20 mL of the algal solutions were sampled from the exposure flasks to determine total *As* concentrations in the cells. After washing the cells twice with sterile Milli-Q water, the 96 h extracellular adsorbed *As* was then washed off for 10 min using ice-cold phosphate buffer of 1 mM K_2_HPO_4_, 0.5 mM Ca(NO_3_)_2_ and 5 mM MES. The gained washing buffer was then retained at 4 °C after filtering it through a 0.45 μm syringe filter to determine the extracellular *As* content [3]. After further 10 min of centrifugation at 4500 × *g*, the settled algal pellets were freeze-dried for further *As* analysis.

The optical density of algal cells was measured at 682 nm wavelength after 0.5, 3, 24, 48, 72 and 96 h of exposure. The growth kinetics were investigated with the exponential model [4] shown in Eq. (1):

(1) $$Ln(X_{t})=N+\mu_{\text{max}}\times t$$

Where $\;X_{t}$ is the optical density (cell mL^−1^) at time *t* (d); *t* is the cultivation time; *N* is a constant; $\;\mu_{\text{max}}\;$ is the maximum specific growth rate (d^−1^).

A nonlinear one-compartment model considering a simultaneous *As* uptake and release was used to describe the measured intracellular concentration of *As* in algal cells for each treatment over time according to the following first-order kinetics:

(2) $${[As}_{int}]=k_{\mu }/k_{e}\times \left[{As}_{med}\right]\times \left(1-e^{k_{e}t}\right)$$

Herein, ${As}_{int}$ (μg g^−1^ dry weight) is the intracellular concentration of *As* in algal cells; ${As}_{med}$ (μg L^−1^) is the *As* concentration in medium assumed to be a constant, and *t* (h) is the time of *As* exposure; *k_u_* (L g^−1^ h^−1^) and *k_e_* (h^−1^) are the *As* uptake and release rate constants for the algae, respectively [5].

Due to the dynamic equilibrium of *As* uptake and release by the algae, the proposed model was only applied if *k_e_* > 0. The modeling was performed with the program Graphpad Prism 7.0 (Graphpad Software). The bioconcentration factor of *As* was calculated as BCF (L g^−1^) = *k_u_*/*k_e_* [6].

#### Release experiment and kinetics model

To determine the *As* release rates from dead cells, 50 mL algal solution of each treatment were taken after 96 h incubation and rinsed with ultrapure water and the aforementioned phosphate buffer. To produce dead cells of *M. aeruginosa*, the samples were heated for 10 min at 50 °C using a waterbath [5,7,8]. Afterwards the treated algae were resuspended in 20 mL sterilized BG-11 media (same with their initial culture conditions) for 8 h, respectively. At 0.25, 0.5, 0.75, 1, 2, 4, 6, and 8 h, 5-mL aliquots were taken from the solutions to determine the algal total *As* concentrations.

The release rate constant for each treatment were evaluated using a simple first-order kinetic (Eq. (3)) [6].

(3) $$Ke=\;-1/t\times \;LnC_{t\;}/C_{0\;}$$

Herein, *C_0_* and *C_t_* represent the intracellular *As* concentration (ng g^−1^) at the start and time *t* (h) of release, respectively; *K_e_* is the release rate constant (h^−1^).

The arsenic partition coefficient (L g^−1^) *K_d_*, between the algae and the aqueous phase were calculated using the formula *K_d_*  = *Ct*/*Cw* (where *Cw* is the measured concentration of *As* in BG-11 medium; μg L^−1^) after 8 h elimination.

### Total arsenic analysis

Total arsenic analysis (*TAs*) of algal cells and the media was determined according to our previously reported method [5]. Briefly, the freeze dried algae were treated by microwave assisted digestion to measure the total *As* amount [9]. We measured the *TAs* concentrations using ICP-MS (Agilent 7500cx, U.S.A) [5]. The signal stability was checked by the simultaneously measured masses of ^72^Ge, and ^103^Rh.

## Additional information

Microalgae are considered as an environmentally-friendly and cost-effective bioremediator for *As*-polluted waters. In the last decade, the bioremediation of metals including *As* as well as their accumulation and uptake dynamics in microalgae have been extensively investigated. In particular, some abiotic factors such as nitrogen (N), phosphorus (P) and pH can impact the *As* metabolism dynamics within the algal cells. Thereby the factors were investigated either separately or in combination under well controlled conditions in laboratory. However, information regarding combined effects on algal arsenate metabolism biokinetics induced by the aforementioned abiotic environmental factors is quite limited.

This eventually warranted to further investigation, improving a practical application of algae for *As* bioremediation. Furthermore, little is known about indirect implications as for instance induced by a secondary *As* release into waters after algal death. This may potentially pose different ecological risks (e.g. via settlement and subsequent biomagnification by benthic organisms) for the aquatic environment compared to a primary *As* contamination. To learn about the combined influence of the environmental factors: N, P, pH and the initial *As*^V^ level (being applied at ambient levels) on the *As*^V^ uptake and release kinetics of *M. aeruginosa*, we investigated the *As* bioaccumulation and efflux dynamics involving algal growth and extracellular and intracellular *As* accumulation as well as *As* release in dead algae. Herein, experimental design of Taguchi method concerned only with the principal effects of selected factors was used in our experiments. The percentage contribution effect of each environmental factor on the investigated metabolic biokinetic (*As* bioaccumulation and release) was thus statistically calculated using an analysis of variance (ANOVA) based on Taguchi method. Our new findings offer valuable insights in how to efficiently utilize algae as bioremediation tool to reduce *As* in contaminated water for practical environment.

## References

[1] Wang Z., Luo Z., Yan C., Xing B. (2017). Impacts of environmental factors on arsenate biotransformation and release in Microcystis aeruginosa using the Taguchi experimental design approach. Water Res..

[2] Yan C., Che F., Zeng L., Wang Z., Du M., Wei Q., Wang Z., Wang D., Zhen Z. (2016). Spatial and seasonal changes of arsenic species in Lake Taihu in relation to eutrophication. Sci. Total Environ..

[3] Levy J., Stauber J.L., Adams M.S., Maher W.A., Kirby J.K., Jolley D.F. (2005). Toxicity, biotransformation, and mode of action of arsenic in two freshwater microalgae (Chlorella sp. and Monoraphidium arcuatum). Environ. Toxicol. Chem..

[4] Dalgaard P., Koutsoumanis K. (2001). Comparison of maximum specific growth rates and lag times estimated from absorbance and viable count data by different mathematical models. J. Microbiol. Methods.

[5] Wang Z., Luo Z., Yan C., Che F., Yan Y. (2014). Arsenic uptake and depuration kinetics in Microcystis aeruginosa under different phosphate regimes. J. Hazard. Mater..

[6] Bradac P., Navarro E., Odzak N., Behra R., Sigg L. (2009). Kinetics of cadmium accumulation in periphyton under freshwater conditions. Environ. Toxicol. Chem..

[7] Chen M., Dei R.C.H., Wang W.X., Guo L.D. (2003). Marine diatom uptake of iron bound with natural colloids of different origins. Mar. Chem..

[8] Miao A.J., Wang W.X. (2006). Fulfilling iron requirements of a coastal diatom under different temperatures and irradiances. Limnol. Oceanogr..

[9] Yin X.-X., Wang L.H., Bai R., Huang H., Sun G.-X. (2011). Accumulation and transformation of arsenic in the blue-green alga Synechocysis sp. PCC6803. Water Air Soil Pollut..

